# Mymou: A low-cost, wireless touchscreen system for automated training of nonhuman primates

**DOI:** 10.3758/s13428-018-1109-5

**Published:** 2018-09-05

**Authors:** James L. Butler, Steve W. Kennerley

**Affiliations:** Sobell Department of Motor Neuroscience, University College London, London, UK

**Keywords:** Cognitive neuroscience, Non-human primates, Automated testing, Animal welfare, Facial recognition

## Abstract

Training nonhuman primates (NHPs) to perform cognitive tasks is essential for many neuroscientific investigations, yet laboratory training is a time-consuming process with inherent limitations. Habituating NHPs to the laboratory staff and experimental equipment can take months before NHPs are ready to proceed to the primary tasks. Laboratory training also necessarily separates NHPs from their home-room social group and typically involves some form of restraint or limited mobility, and data collection is often limited to a few hours per day so that multiple NHPs can be trained on the same equipment. Consequently, it can often take a year to train NHPs on complex cognitive tasks. To overcome these issues, we developed a low-cost, open-source, wireless touchscreen training system that can be installed in the home-room environment. The automated device can run continuously all day, including over weekends, without experimenter intervention. The system utilizes real-time facial recognition to initiate subject-specific tasks and provide accurate data logging, without the need for implanted microchips or separation of the NHPs. The system allows NHPs to select their preferred reward on each trial and to work when and for as long as they desire, and it can analyze task performance in real time and adapt the task parameters in order to expedite training. We demonstrate that NHPs consistently use this system on a daily basis to quickly learn complex behavioral tasks. The system therefore addresses many of the welfare and experimental limitations of laboratory-based training of NHPs and provides a platform for wireless electrophysiological investigations in more naturalistic, freely moving environments.

Training nonhuman primates (NHPs) to perform cognitive tasks in the laboratory is a time-consuming process with important limitations. Upon arrival in a facility, NHPs must first acclimatize to their new surroundings and the experimental staff. The NHPs must then learn to enter a testing chair (sometimes via pole and collar) to transport them to a testing setup in the laboratory, which necessarily separates the NHP from the comfort of their home environment and peer group. Once in the laboratory, experimental tasks may require NHPs to indicate behavioral responses via joysticks, levers, buttons, or other chair-mounted devices, based on sensory information from a video monitor or speaker mounted some distance away. These abstract mappings between peripheral sensory cues and chair-mounted devices can be difficult to learn. Even once task training progresses, NHPs are typically only trained for 1–2 h per day in a laboratory session, before either becoming tired or sated with reward, or so that the experimenter can train other NHPs with the same laboratory equipment. These are important considerations, as it can often take a year to train a naïve NHP to perform a complex cognitive task.

Laboratory-based training also has important welfare considerations for the NHP. NHPs are commonly transported and tested in the laboratory in testing chairs that impose some restrictions on mobility (McMillan, Bloomsmith, & Prescott, 2017). Such chair restraint, potentially in combination with separation from the peer group, can increase stress levels in NHPs (Shirasaki et al., 2013). Finally, unlike in their home room, where NHPs can typically forage for resources or explore their environment when desired, during laboratory-based training the experimenter controls when the NHP is tested, and hence when food or fluid rewards are available. If NHPs are tired, sated, or otherwise unmotivated when the experimenter schedules testing, the testing conditions may not be optimized.

Given these limitations, there is growing interest in developing automated systems that would allow behavioral training in the home environment. Fully automated testing systems have been used in external environments that NHPs can freely access from their home environment (Claidiere, Gullstrand, Latouche, & Fagot, 2017; Fagot & Bonte, 2010; Fagot & Paleressompoulle, 2009; Fizet et al., 2017; Gazes, Brown, Basile, & Hampton, 2013; Libey & Fetz, 2017). Other automated training systems can be attached to either the NHP’s home cage or adjacent cage environments (Calapai et al., 2017; Fizet et al., 2017; Truppa et al., 2010; Tulip, Zimmermann, Farningham, & Jackson, 2017), and other unpublished systems may exist using open-source NHP software (e.g., MWorks, MonkeyLogic) that could be adapted to the home cage. However, the currently published systems all have limitations. First, because accurate subject data logging is required, some automated behavioral training systems require separation of the NHP from their cage mates (Mandell & Sackett, 2008; Truppa et al., 2010; Weed et al., 1999), and thus share the same disadvantages as the laboratory-based training described above. Those systems capable of behavioral training of group-housed NHPs rely on either the use of invasive radio-frequency identification (RFID) implants (Fagot & Bonte, 2010; Fizet et al., 2017; Gazes et al., 2013; Tulip et al., 2017) or labor-intensive manual sorting of footage of the NHPs using the system (Calapai et al., 2017). Furthermore, some of these systems require specialist components that are expensive and hard to source (Calapai et al., 2017; Fagot & Bonte, 2010).

To overcome these issues, we introduce the Mymou system (Greek for “monkey,” pronounced *my-moo*), a low-cost, open-source, fully automated wireless touchscreen training system constructed from off-the-shelf components. The automated device can run continuously all day, including over weekends, without supervision or intervention from the experimenter. It utilizes a real-time facial recognition algorithm that can initiate subject-specific tasks and provide accurate data logging. This is a novel feature for automated training of NHPs, and allows for task training without the need for separation of NHPs or RFID implantation. Complex tasks can be written with just basic knowledge of the Java programming language, and all of the requisite code is publicly available. The system allows the training of NHPs immediately after their arrival at the facility, allows NHPs to work when and for as long as they want, and can analyze task performance and adapt the task parameters in real time in order to expedite training.

## Method

**Figure 1.**
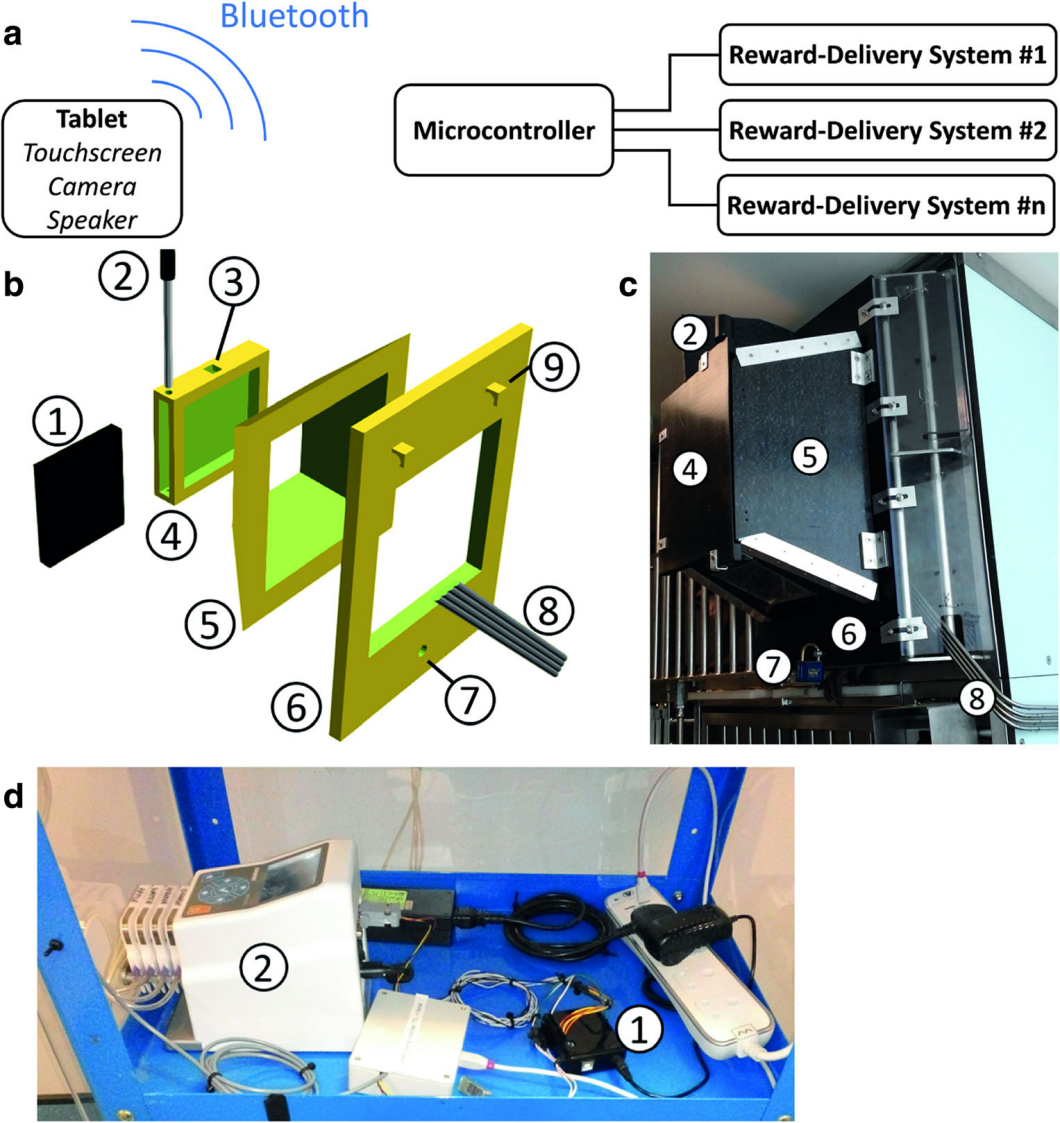
Components of the Mymou system *A*, The system is fully wireless and uses Bluetooth to communicate with reward delivery systems. ***B***, 3D schematic of the holder that was used to secure the tablet to the outside of the cage. Consisting of 1) tablet, 2) metal locking pin, 3) hole for optional charging cable, 4) tablet holder, 5) sloped funnel, 6) front panel, 7) hole to lock the holder to the cage, 8) reward tubes, 9) hooks to mount holder to cage. ***C***, Photo of the holder depicted in ***B***. Numbers correspond to the same items in ***B***. ***D***, Photo of the microcontroller (1) connected to a 4-channel peristaltic pump (2)

The Mymou system is designed to allow behavioral testing of NHPs in their home room using low-cost, lightweight, and accessible components. The Mymou system is split into two separate components, the behavioral testing unit (BTU) and the reward-delivery interface (RDI). The BTU communicates with the RDI wirelessly over Bluetooth, and therefore the two systems are physically independent of each other (Fig. 1A). The total cost for the Mymou system is £385, with the main costs being the electronic tablet and tablet holder (Table 1, prices accurate as of 2 July 2018).

**Table 1:** List of parts needed for the Mymou system

Type		Component	Price (£)	Source
Hardware	RDI	Arduino Uno Microcontroller	19	Amazon
		Bluetooth extension	7	Amazon
		Type A to B USB cable	4	Amazon
		USB mains power adapter	7	Amazon
	BTU	Android tablet	171	Amazon
		Tablet mounting device	177	theplasticshop.co.uk, RS Components
Software	RDI	Arduino IDE	Free	Arduino
	BTU	Android Studio	Free	Google

Not included is a reward-delivery system. The tablet mounting device was designed by an in-house engineer.

### Hardware

#### Behavioral testing unit

The BTU consists of two parts: an Android tablet, which is used for delivering the behavioral task and communicating with the RDI, and a holder to secure the tablet against the side of the home cage (Figs. 1B–1C). For the BTU, a Samsung Galaxy Tab A (2016, 16 GB, 10.1 in.) running the Android operating system Marshmallow 6.0.1 was used. Any device running any of the Android versions 6.0.0 and above can be used, as long as the unit has Bluetooth capabilities and a front-facing camera. However, no other OS versions have been tested, and some compatibility issues might occur that, although likely minor, would require correcting before the system could be successfully deployed. Tablets using Android OS are recommended, since all the behavioral and experimental routines are written in this language.

A holder made from acetal plastic was made by an in-house engineer to secure the tablet to the front of the cage. The design was adapted from Calapai et al. (2017), using a square frustum to enclose the unit while affording the subjects generous access to the tablet through the bars of the cage (Fig. 1B). The holder could be left mounted on the cage, and the tablet easily slid in and out as needed. A charging port was also present on the holder, which allowed for the tablet to be charged while in use, although the battery life on the tablet (> 12 h) means that the system can be used wirelessly throughout the day. A 3-D model of the holder, to scale, is available on our Github page (https://github.com/jamesbutler01/Mymou), which can be used to replicate the different parts as required.

#### Reward-delivery interface

The RDI is a mediator between the BTU and the reward-delivery system (RDS). An Arduino Uno microcontroller board (Adafruit Industries, New York, USA) was used, which is both inexpensive and easily accessible to those without a background in electronic engineering. An additional Bluetooth module is connected to the microcontroller, allowing it to receive commands from the BTU and control the connected RDSs as desired. The microcontroller can control up to 11 RDSs/channels simultaneously, although only four were used here. It communicates using conventional digital signals, and any RDS that can be controlled via 5-V digital signals will work with the RDI. Here, a four-channel peristaltic pump (Reglo ICC, Ismatec, Wertheim, Germany) was used to independently deliver different fluid rewards. The system has also been used with food pellet delivery systems (Med Associates Inc., Fairfax, USA).

### Software

The software for the Mymou system is written in three languages, but basic knowledge of only one is sufficient to operate the Mymou system. Java is used to design the behavioral tasks to be run on the BTU, C++ is used to control the RDI, and Python is used to train the facial recognition system. The latest versions of all our scripts can be found on our Github page (https://github.com/jamesbutler01/Mymou), where they are regularly updated. The project is fully open-source, and contributions from other users are encouraged. Any issues with the Mymou system can also be brought to our attention on this page.

#### Java (BTU)

The BTU is controlled by an application built in Android Studio using the Java programming language. This consists of two back-end modules, which control the Bluetooth and the facial recognition functions, and a front-end module for the behavioral tasks themselves. The back-end modules do not require any input from the experimenter. The front-end module requires programming to design whatever behavioral task is desired. Many of the functions required for behavioral tasks are already provided on our Github page, such as scripts to log task data, deliver rewards, and to animate task objects. Only relatively small amounts of code are needed for complex task designs.

The device operation is fully customizable, specifying the times of the day that subjects are able to use the system. This can also be modified by the day as well, for example to restrict access on the weekends. During inactive periods the device enters a dormant state displaying a black screen with a low brightness that does not register any touches from the subjects. During active periods the screen returns to full brightness and displays the task, alerting subjects that they can now use the device. At the end of the testing day, a timer can trigger the reward pump to flush water through the juice lines to clean the system.

The BTU saves all data to the device’s internal memory, but the software can easily be altered so as to save data to the external SD card, if required. Data are stored in a new folder each day, named for the date of training (e.g., 20180226). Within this folder, a folder named “i” (for *images*) stores all photos taken by the camera during the day, which can be used for manual subject verification. These images are also converted to numerical arrays and stored in a folder named “f” (for *floating point array*), which are used by the facial recognition system. Finally, a text file (also named for the date of training) that contains all the behavioral events from that day is also created in the folder. In this text file, there is one row per task event (e.g., NHP press, reward delivery), with as many columns as required to contain all the relevant information (e.g., event timestamp, timestamp of the photo that corresponds to the event, outcome of the trial, etc.). Each image or float array generated is saved under the name of the time at which the photo was taken (in the format HHmmss_iii, where H = hours, m = minutes, s = seconds, and i = milliseconds), allowing one to match up events in the log file with the corresponding images in the image folder. At midnight, the device automatically starts saving events to the new date, allowing the program to store data across multiple days without issue. The data can then be copied across to a computer for offline analysis when needed. No automated backup system is provided, so it is advised to transfer the data from the device regularly. However, many third-party applications could be used to automatically back up the data, though data security should be considered.

The BTU can be configured to email information to the experimenter as often as is desired, allowing for remote monitoring of the task. This includes both information about the current task, such as the number of trials completed or the amount of reward given, information about task performance (e.g., reaction times or accuracy by condition), or information about the unit itself, such as the amount of battery remaining. These emails can also be set to be triggered rather than sent periodically—for example, to warn the experimenter if the battery is getting low or if the device has lost its Bluetooth connection to the RDI.

One caveat of automated training is that the experimenter is not around to observe any errors that may occur. The BTU also supports extensive error monitoring. In its default state, the unit logs any errors for the experimenter to review later (or they can be emailed in real time). In the meantime, if an error occurs, the device automatically restarts the task, to allow training to continue without the need for experimenter intervention.

#### C++ (RDI)

A concise, custom-written C++ script executed in the Arduino IDE is used to control the RDI. The script requires no alterations from the experimenter and can be directly loaded onto the microcontroller as provided. It functions to receive event codes sent from the BTU via Bluetooth, and activates or deactivates the different RDS channels appropriately. In general, one unique event code activates a particular channel, and a second unique event code deactivates the channel. Table 2 details all the event codes and the digital channels of the microcontroller that they control.

**Table 2:** List of event codes the RDI responds to and the effects they have

Event Code	Channel	Value
0	All	Low (0 V)
1	2	High (5 V)
2	2	Low (0 V)
3	3	High (5 V)
4	3	Low (0 V)
5	4	High (5 V)
6	4	Low (0 V)
7	5	High (5 V)
8	5	Low (0 V)

It is important to note that the RDI is designed to act as a passive mediator between the BTU and the RDSs. It therefore possesses no fail-safes and relies on the BTU to adequately regulate RDS activity.

#### Python (facial recognition training)

The supervised training of the artificial neural network (ANN) used for facial recognition is initially trained using Python on a PC. This was done because of the high computational demand that this training places on a processor, which is better suited to faster desktop CPUs than to the slower CPUs present in Android tablets. This script requires minor input from the experimenter, simply to point it to the location of the subject-labeled dataset to be used for the supervised learning.

The supervised training of the ANN proceeds as follows. First, a previously collected dataset of labeled photos of each subject is required. A preprocessing script sorts through the labeled photo files, appending them into a single 2-D matrix (one row per image). This matrix is then fed into the main script, which randomly shuffles the files and splits them into a training set and a test set. The ANN consists of an input layer with one neuron for each pixel of the input images, a fully connected hidden layer whose size can be specified (in our case 20 worked best), and a fully connected output layer of one neuron per subject (in our case two to four). Gradient descent is used to train the weights of each layer, such that each of the output neurons only responds to images of a single subject. Various hyperparameters can be modified by the experimenter to optimize the performance of the ANN, although in our experience the system worked well with little or no adjustment of the hyperparameters. These include the number of training iterations, the size of the hidden layer, and the learning rate. After training is completed, three files are produced that contain the mean and variance of the training data (needed for normalizing future inputs to the system), the optimized weights for the hidden layer, and the optimized weights for the output layer. These files then need to be simply transferred to the BTU, where they are used for online facial recognition.

### Security

Due to the fact that the tablet stores potentially sensitive information about experimental animals, great care was taken to ensure the security of this information. The Android operating system is fully encrypted and password protected, ensuring that any unauthorized users that gain physical access to the device will not be able to access the data itself. However, while the task is running, anyone who gains access to the device will be able to access the photos and other data stored on the device. With the exception of the email reporting function (which can be disabled), the device can be run without an internet connection, providing immunity to any remote unauthorized access. In any case, we recommend consulting your information technology specialist to ensure that the data are secure.

### Animals

The Mymou system was used to train two male rhesus monkeys (*Macaca mulatta*), 3 and 4 years of age at the start of the project, over a period of 182 sessions spanning 10 months. All training sessions were conducted with both subjects in their home cage, and at no point were the subjects separated for individual training. The system was left on for up to 12 h in a session, with subjects being left free to play at their own pace. Neither of the subjects had implanted devices.

For the first 41 experimental sessions (10 weeks), subjects were given ad lib access to water outside of when they were given access to the Mymou system (minimum 14 h of water access a day). For a further 36 sessions over 6 weeks, the subjects were given ad-lib access to water for 159 ± 11 min (minimum 90 min) outside of when they were given access to fluid via the Mymou system. On days when no training occurred, the subjects had ad lib access to water. For the remaining 123 sessions over 22 weeks, subjects received the majority of their fluid from the rewards on the Mymou system and were only given a water supplement at the end of the day if they failed to obtain their daily minimum fluid requirement. All experimental procedures were approved by the Home Office and the Local Ethical Procedures Committee and were carried out in accordance with the UK Animals (Scientific Procedures) Act.

To assess the individual juice preferences of the two subjects, the following four liquids were offered, each diluted with water: Orange squash (Robinsons Orange, Britvic Soft Drinks Ltd, Dublin, Ireland), apple juice, pineapple juice, and blackcurrant squash (Ribena blackcurrant, Lucozade Ribena Suntory Ltd, Uxbridge, UK). Water was also often offered as one of the reward types.

## Results

### Using the Mymou system

The system was used for a total of 182 sessions over a period of 10 months. Even when not in use, the system was normally left in place in the home cage in a dormant state. Two tablets were used in tandem and swapped with one another whenever the behavioral data needed to be collected or the task needed to be altered. This allowed the tablets to be swapped in a matter of seconds, without having to wait for the device to be charged or for data to be transferred. At the end of the 10 months, there was no observable damage to either of the tablets used or to the external tablet holder, demonstrating that the Mymou system is robust and can be used for long periods of time.

The tablet could easily last a full experimental day, losing approximately 50% battery over a 10-h period. In its dormant state when not in use (such as when left overnight), it used only approximately 10% of its maximum battery capacity in the same 10-h period. Therefore, when used wirelessly, the system could comfortably last for a day and a half of training, intersected with an overnight period, before the experimenter needed to charge the device. We primarily used the Mymou system with the tablet plugged in to charge. In this state, and coupled with the automated task timing, the device was able to run indefinitely without any attention from the experimenter.

In our facility, cages are hosed down and cleaned at least once a week. During this time the electronic tablet could be slid out of its holder in a matter of seconds by untrained staff and replaced after the washing had finished. There was no need to restart the device after this had occurred, and the tablet continued to function no matter how many times it was removed from and replaced in its holder.

### Behavior

During initial exposure to the Mymou system, the experimenter held the tablet and gave the subjects food rewards in exchange for pressing the screen. After doing this for five consecutive sessions of 10 min, the subjects knew to interact with the tablet for reward, and from this point onward subjects interacted with the tablet in their home cage via the tablet holder shown in Fig. 1.

**Figure 2.**
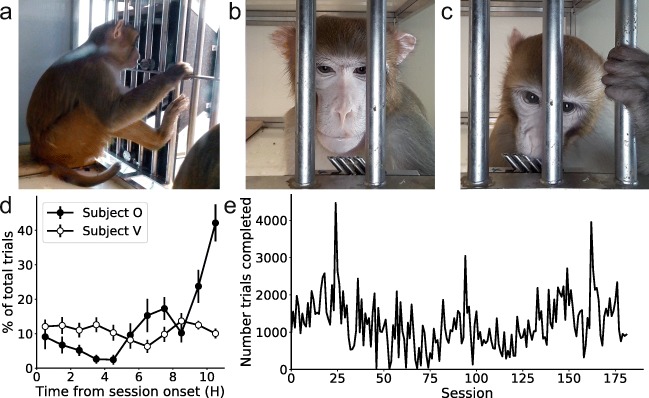
The Mymou system in use *A*, One of the non-human primates using the device. *B*, A selfie taken by the device triggered by Subject O initiating a trial. The stainless steel spouts used for delivery of a juice reward can be seen at the centre bottom. ***C***, An example of subject V receiving reward while attend-ing to the task on screen. ***D***, The distribution of when each NHP would complete trials throughout a single session (n = 7). Error bars represent standard error of the mean. ***E***, The number of trials completed between both NHP’s across all sessions. Note that the tasks they were doing varied dramatically over this period

The design of the tablet holder allowed subjects to comfortably interact with the device for long periods of time (Fig. 2A). The front-facing camera was able to capture clear images of the subjects while they engaged with the device (Fig. 2B), and subjects were able to receive juice rewards while still attending to the task on screen (Fig. 2C).

Subjects would engage with the system throughout the day; however, there was a marked difference in the routines of the two NHPs (Fig. 2D). Subject V consistently interacted with the device throughout the entire session. Subject O, however, increased his participation as the session progressed, presumably as his thirst accumulated across the day. Different NHPs therefore prefer to work or prefer fluid at different times of the day, and the Mymou system accommodates this.

Both subjects engaged with the Mymou system on a daily basis, completing 1,239 ± 52 trials per session between them (Fig. 2E, *n* = 182; data are collapsed across subjects because single-subject data were not available for earlier sessions, due to the facial recognition package still being developed). There were large fluctuations in participation on a session-by-session basis (Fig. 2E); however, over the full time course, 36 different task versions were given to the subjects, as they progressed to more complex task designs with higher cognitive demands. There were large differences in the lengths of these different trials, with some requiring just one choice per trial, and others requiring subjects to make five consecutive correct choices for a completed trial. Therefore, below we describe data from one specific time period of 18 sessions, over 21 days in which there was no change in the task, to serve as a detailed example of training NHPs with the Mymou system.

### Learning with the Mymou system

**Figure 3.**
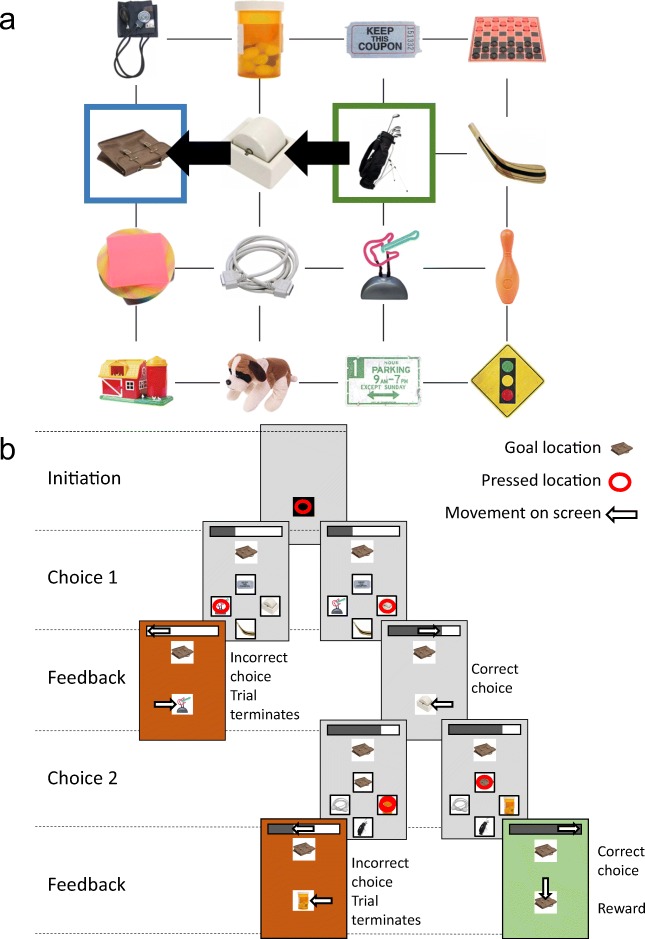
A task that was used on the Mymou system *A*, Subjects were taught a network of 16 stimuli arranged in a 4x4 grid, with each stimulus being associated with its neighbours. From this network 68 unique paths of distance two were present. For example starting at the green highlighted stimulus and ending at the blue highlighted stimulus. ***B***, Schematic of the task that was used to teach the associative network shown in ***A***. When a trial is intitiated a goal location and four possible options to choose from are presented (choice 1). Upon making a choice animations then provide intuitive, engaging feedback to the subject. In total two correct choices were needed to receive a reward. Getting either choice wrong results in trial termination and a timeout of 1-3 seconds

In these tasks, we were interested in associative learning and the capabilities of NHPs to learn complicated associative networks of stimuli. To test this, we used a network of 16 different stimuli arranged in a 4×4 square grid (Fig. 3A). The stimuli were randomly taken from the image database provided by Brady, Konkle, Alvarez, and Oliva (2008). Within this network, each stimulus has two to four associated neighbors, resulting in a total of 68 associations across the entire network. The goal was for the subjects to learn how all the stimuli were connected, so that they could navigate between linked stimuli to reach a goal stimulus. Asking subjects to navigate to targets multiple associations away greatly increased the cognitive demands of the task, because subjects had to plan paths through the network in order to reach a target location.

The task design using the Mymou system is shown in Fig. 3B. Subjects had previously learned the concept of a progress bar at the top of the screen, which indicated how many remaining correct choices were required in order to reach a goal and obtain reward. Subjects would initiate a trial by touching a neutral stimulus (a black square) on the screen. Subjects would then be presented a “goal” location stimulus, along with the two to four stimuli that neighbored their current position in the grid (which was not shown but could be inferred). Subjects were required to choose one of these stimuli. The chosen option then moved to the center of the screen while the other stimuli disappeared. If a stimulus in the direction of the goal stimulus was chosen, the progress bar filled up proportional to the remaining steps to reach the goal. If the choice was to a stimulus away from the goal stimulus, then the progress bar drained to empty and the screen turned red to indicate an incorrect choice and termination of the trial. The subject had to make successive choices of stimuli until the goal stimulus was reached and reward delivered. The number of choices required was dependent on the distance between the starting state and goal stimulus.

**Figure 4.**
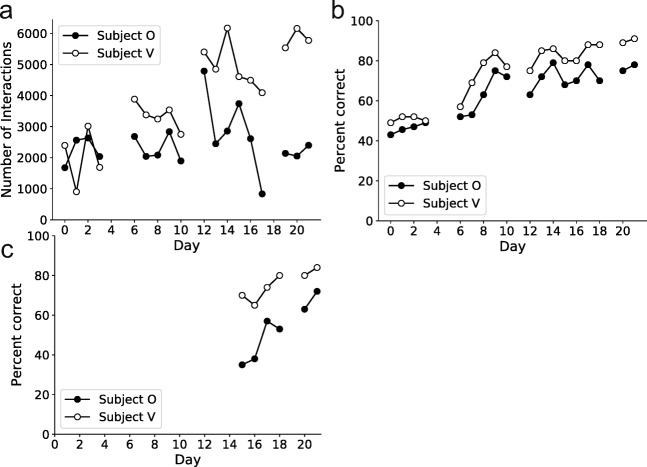
Subjects learnt effectively on the Mymou system *A*, Total number of interactions with the system over 18 training sessions across a 3 week period. ***B***, Daily performance over the three week period on the task at navigating two transition problems. ***C***, Same as in ***B*** but for when given harder problems three transitions in length

Throughout the duration of training, the subjects continually engaged with the task (Fig. 4A). Over the course of training, Subject V steadily increased the number of trials completed per session, likely due to the decreased effort-to-reward ratio as his performance increased. Both subjects learned the network of stimuli over the course of training, increasing the percentage of times they were able to correctly navigate two transitions across the stimuli (subject O increased from 43% correct to 78% correct, and subject V increased from 49% to 91%; Fig. 4B). Later in training, harder problems (e.g., distances of three transitions between the starting and goal locations) were presented to the subjects. The subjects quickly learned to solve these problems, too, exhibiting a steady increase in correctly performed trials over just six training sessions (subject O increased from 35% to 72% correct, and subject V increased from 70% to 84% correct; Fig. 4C). The Mymou system was therefore effective at teaching NHPs very abstract and complex cognitive tasks. Concurrently, both NHPs were also trained on different tasks in the laboratory using chair-mounted joysticks. Thus, using the Mymou system in the home cage did not appear to diminish an NHP’s willingness to enter a testing chair and learn laboratory-based tasks for reward.

### Facial recognition

**Figure 5.**
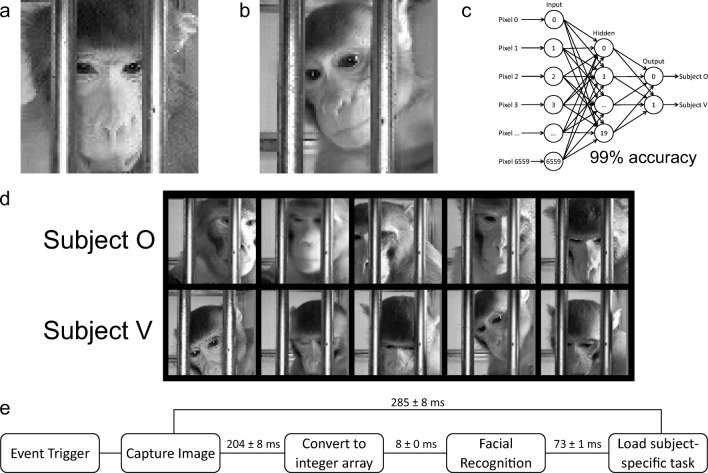
Online facial recognition *A*, A cropped selfie of subject O that was used for identification of the subject. ***B***, A cropped selfie of subject V that was used for identification of the subject. ***C***, Schematic of the neural network that was used for facial recognition. Each circle represents a neuron and each arrow represents a weight that modulates the input from the upstream neuron. The network was able to correctly identify subjects with 98.89% accuracy. ***D***, Examples of images correctly identifed by the ANN of subject O (top row) and V (bottom row). ***E***, Timeline of the online facial recognition process, data taken from 10 iterations of the process

The facial recognition began by first training an ANN on a set of subject-specific images. A total of 6,512 facial images were taken over 13 days while the subjects used the Mymou system (Figs. 5A and 5B). From manual observation, it was clear that the position of the face of both NHPs was always in the center of the image, with the edges of each image containing no useful information for facial recognition. To reduce the processing time of the ANN, the edges of the full-size images (176 × 144 pixels) were removed, leaving only the center 80 × 82 pixels. The amount of cropping can easily be adjusted in the provided source code, although to avoid retraining the ANN, this should be kept generous, to ensure that the entire face is visible, since the ANN must be trained on images of the same size. This cropped image is then saved to the device, in case offline manual verification is required. The image is then converted to grayscale and flattened into a 1-D array of length 6,560 and saved to the device for use with the ANN. The images were split into two equally sized groups, a test set and a training set, the latter of which was used to train the ANN.

The structure of the network was an input layer of 6,560 neurons, followed by a hidden layer of 20 neurons, and finally an output layer of two neurons (Fig. 5C). The network was fully connected, resulting in 131,200 weights (6,560 × 20) for the hidden layer, and 40 weights (20 × 2) for the output layer. Gradient descent was used to tune these weights over 60 iterations of the complete training dataset. The test dataset was then used to check the accuracy of the ANN, which distinguished between the two subjects with 98.89% accuracy.

The ANN was capable of correctly identifying each subject despite variations in head position and head orientation or blurred facial features in the images. Some examples of correctly identified photos are provided in Fig. 5D. Manual verification of the ANN confirmed its accuracy.

The trained weights were then loaded onto the Mymou system and used for real-time facial recognition. The ANN was then used each time an image was captured to return the predicted identity of the subject in the image. Upon an initial touch event by a subject, the Mymou system would take 204 ± 8 ms to capture an image, 8 ± 0 ms to convert the image to a flattened 1-D array, and 73 ± 1 ms to run the array through the ANN and return the predicted subject identity (285 ± 8 ms in total, *n* = 10; Fig. 5E).

To assess the effectiveness of the ANN on a larger population, another two male rhesus macaques 6–7 years of age were trained to use the Mymou system. We first introduced them to the Mymou system by manually rewarding them with a food reward for pressing the tablet screen for 10 min a day for 5 days. They were then left each in the cage with the Mymou system for 2 h. During this time, the two NHPs quickly learned that the spout dispensed juice upon touching the tablet, and they engaged with the tablet 343 and 824 times each. We then pooled the images across the four NHPs and generated a training batch of 300 images per subject, 240 of which were used to train the ANN. When tested on the remaining 60 images per subject, the ANN successfully predicted the subject identity with an accuracy of 98.99%. Furthermore, when the training dataset was reduced from 240 to just 40 images per subject, the ANN could still predict the subject identity with an accuracy of 93.75%. The ANN was therefore capable of highly accurate facial recognition within a group of four NHPs, and needed only a few images for training to reach a high level of accuracy.

The predictions generated by the ANN was appended to the behavioral data as it was stored, resulting in subject-specific data that could be easily analyzed afterward. The ANN could also be used online to direct training in a subject-specific manner, due to its short latency (Fig. 5E). The ANN is therefore fast enough to initiate subject-specific behavioral tasks, provides accurate behavioral data logging without the need for embedded microchips, and allows for behavioral training in group-housed environments without the need to separate subjects.

### Reward preferences

Another feature of the Mymou system is the ability to control multiple RDSs simultaneously. We used this feature to allow subjects to choose rewards on a trial-by-trial basis, which allowed us to explore how reward preferences changed both within and across behavioral testing sessions. We used a four-channel peristaltic pump to deliver four different fluids within a single training session. At the end of a successful trial, an additional screen presented four unique images, each associated with a specific juice reward on offer. This allowed subjects to explore the different types of rewards available and to switch between rewards if they became sated with one option.

**Figure 6.**
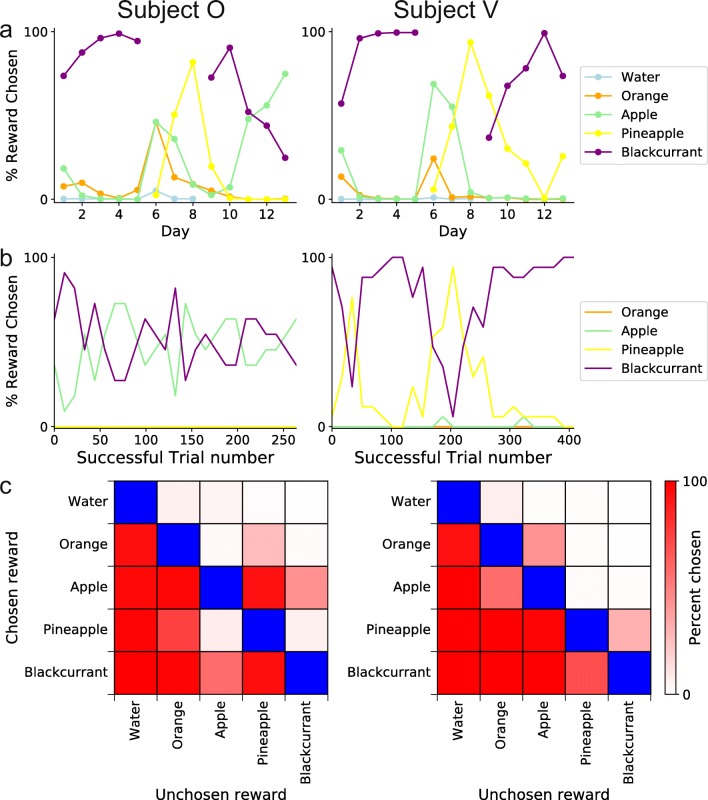
The Mymou system can control multiple reward delivery systems *A*, After each success trial subjects were given a choice of four different juices to pick as a reward. The amount they picked each different juice across all days tested is displayed. ***B***, Juice preferences over a single day, taken from day 11 in ***A*** to show the switching behaviors that were sometimes exhibited. ***C***, Matrix of overall juice preferences for each subject, calculated from all the data shown in ***A***

We tested a total of five different fluid rewards (water, orange, apple, pineapple, and blackcurrant), with different combinations of four of the rewards being offered in individual sessions. Preferences varied over the course of the experiment and between the two subjects (Fig. 6A). For example, subject O originally preferred blackcurrant to apple for several sessions, but in later sessions this subject developed a preference for apple juice. Although in most sessions subjects would stick to one preferred option, sometimes subjects were less decisive and switched between two or three different options (Fig. 6A). In sessions in which subjects preferred two or more rewards (e.g., Day 11 of Fig. 6A), the nature of switching between rewards was variable; subject O often alternated between two options (Fig. 6B, left), whereas subject V would switch at a lower frequency, sampling the same option for longer periods of time before switching to a different reward (Fig. 6B, right). Both subjects also exhibited a clear hierarchy in reward preferences between all offered juices, with blackcurrant and apple juice being the two most preferred rewards (Fig. 6C).

The ability of the RDS to accurately dispense juice was assessed by weighing the amount of liquid perfused for different durations of pump activation. Activating the pump for 1,000, 1,500, 2,000, or 2,500 ms perfused 0.18 ± 0.04, 0.29 ± 0.06, 0.45 ± 0.03, and 0.59 ± 0.05 g of juice, respectively (*n* = 10 in each case, with data presented as mean ± standard deviation). Thus, although the accuracy of reward delivery will depend on the reward device used, the RDS is capable of dispensing small amounts of liquid with high precision.

The Mymou system allows subjects to choose between preferred rewards on a trial-by-trial basis. This can be used to build a comprehensive picture of an individual’s reward preferences, which can run concurrently to the experimenter’s main task. Allowing the subject to choose a preferred reward on a trial-by-trial basis reduces reward-specific satiety, which may motivate performance and facilitate behavioral training.

## Discussion

We have developed a low-cost, open-source, entirely automated in-cage testing unit that can be easily used with off-the-shelf components. The device was used to train NHPs to perform demanding cognitive tasks within their home room rather than in the laboratory. The system allows training to proceed across the entire day, with accurate data logging, and allows the subject to work for rewards at its own pace and leisure. We consider the Mymou system to be a refinement (according to the 3Rs principle: Macarthur Clark, 2018; Prescott & Lidster, 2017; Russell & Burch, 1959), relative to laboratory or other in-cage training methods, and we highlight its advantages below.

### Advantages over laboratory-based training

With laboratory-based training, NHPs must be trained to enter a testing chair (which may involve the use of a pole and collar), acclimate to leaving their home environment, and learn how to use chair-mounted devices (levers, buttons, etc.) to interact with the task. This process can take months before NHPs are ready to perform the primary experimental tasks. With the Mymou system, NHPs can begin learning tasks immediately upon arrival at the facility. Experimenters can capitalize on NHPs’ natural curiosity to prompt them (via sounds or stimuli presented from the tablet) to touch the screen and explore different rewards. In our case, NHPs were using the system without experimenter intervention after 5 days of training.

The Mymou system is fully automated and can be left running for as long as the rewards are kept topped up. This is advantageous to the NHPs, who are then able to work at their own pace (including taking breaks to eat, drink, or play between trials) throughout the day, instead of having the experimenter control when they work, and hence when food/fluid rewards are available. This flexible work routine seeks to minimize satiety, boredom, or fatigue during the training process. In certain cases, such as for electrophysiological experiments, it may be desirable to train NHPs to work in condensed periods rather than in a leisurely fashion throughout the day. The device can be configured to only turn on for a certain time window each day, to acclimatize NHPs to this kind of work schedule instead.

### Advantages over other automated training systems

There are several excellent automated systems for the training of NHPs, either in the home cage or in adjacent environments (Calapai et al., 2017; Fagot & Bonte, 2010; Gazes et al., 2013; Tulip et al., 2017). Although each system has its own strengths, the Mymou system has four main advantages over the group of current automated training systems.

First, a limitation of current systems is that accurate subject identification and data logging requires either invasive RFID implants (Fagot & Bonte, 2010; Gazes et al., 2013; Tulip et al., 2017) or labor-intensive manual sorting of footage (Calapai et al., 2017). As we have shown, NHPs use the Mymou system for thousands of trials a day, an advantage when training NHPs on complex tasks, but this also makes subject identification and data logging via manual image sorting impractical. The Mymou system uses real-time facial recognition, which has not previously been used in automated systems for training NHPs. The system was capable of near-perfect (99%) accuracy, despite large variations in the position and angle of each NHP’s head, making it suitable to use in a home room setting where NHPs have full mobility during training. This eliminated the need for any manual sorting of photos in order to identify which NHPs were using the system. In the case that NHPs are single-housed or receive RFID implants as a matter of routine or in conjunction with other surgeries, the full implementation of the Mymou system (i.e., the facial recognition for implementing subject-specific tasks and accurate data logging) may be unnecessary, and a simpler device may perform just as well.

Artificial neural networks are the leading technology for (human) facial recognition (Parkhi, Vedaldi, & Zisserman, 2015; Schroff, Kalenichenko, & Philbin, 2015; Taigman, Yang, Ranzato, & Wolf, 2014), which can distinguish between populations of individuals many orders of magnitude beyond the largest monkey colony. We have used an ANN for facial recognition for pair-housed NHPs, one of the most common housing strategies for NHPs in scientific experiments worldwide (DiVincenti & Wyatt, 2011; Hannibal, Bliss-Moreau, Vandeleest, McCowan, & Capitanio, 2017; National Centre for the Replacement, Refinement and Reduction of Animals in Research, 2017; National Research Council, 2011; Scientific Committee on Animal Health and Welfare, 2002; Truelove, Martin, Perlman, Wood, & Bloomsmith, 2017). However, the parameters of an ANN can be adapted to a more sophisticated level for use in larger social groups, such as by adding convolutional layers, to improve the ANN’s performance, if necessary. Alternatively, other facial recognition packages have been developed for use in large NHP groups (Witham, 2018), which could be used with the Mymou system in place of the ANN.

Second, the facial recognition algorithm accurately identified the NHP within 300 ms of a screen touch. This real-time subject identification allows the system to generate subject-specific parameters, ideal for pair- and group-housed NHPs. For example, the Mymou system can load different tasks, make different rewards (food or fluid) available, activate different manipulanda (joysticks, buttons, or other Mymou systems), all within 300 ms of initiating a trial, such that NHPs at very different stages of training, or on very different tasks or task parameters, can all use the same system. Coupled with programmable timers and a long battery life, the Mymou system allows for fully automated training over an entire day, including weekends, without requiring an experimenter to initiate tasks, adjust settings, or separate/interact with NHPs in their home environment.

Third, the Mymou system is also capable of analyzing behavioral performance as the subject performs the task. This allows the system to switch tasks or conditions on the fly or make the system inaccessible, if a set performance criterion has been met. The Mymou system can also email the experimenter regular performance updates for each NHP. This is useful for keeping track of any individuals that may be struggling with the current task design, and it also allows the experimenter to keep track of how much reward each subject has received.

Finally, the Mymou system is affordable and can be easily installed in traditional home room environments. Whereas some of the leading automated training systems have been used in specialized testing booths or cages either outside, or attached to, their primary home cages (Calapai et al., 2017; Fagot & Bonte, 2010; Gazes et al., 2013; Mandell & Sackett, 2008; Truppa et al., 2010; Tulip et al., 2017), the Mymou system allows for training NHPs in their home room environment and works extremely well in pair-housed environments. Moreover, some of the more sophisticated automated systems require expensive specialist components that are hard to source and assemble (Calapai et al., 2017; Fagot & Bonte, 2010). The Mymou system, in contrast, costs under £400 and is made from commercially available components, and we provide all of the source code and instructions for assembly and use.

### Limitations of the Mymou system

The Mymou system has a few functional limitations as it is currently configured. First, there is no remote monitoring of the device, apart from text-based email alerts, and users cannot remotely activate or deactivate the device (though activation/deactivation can be scheduled in the software). Second, no automated data backup system is provided, so failing to manually back up the device at regular intervals risks data loss in the event the device fails (although this never occurred for us in over 10 months of use). Both of these aforementioned limitations can be solved using third-party applications (e.g., Dropbox, TeamViewer), but given the sensitive nature of the data, careful consideration should be taken before giving third-party applications access to the device. It should be possible to back up the data via closed-network wifi, although we have not explored this option.

Another limitation is that the BTU–RDS relationship is currently one-directional, so it cannot receive information from the RDS, such as the status of the juice reservoir. This currently requires user monitoring in order to ensure that the juice reservoir is never depleted when the system is active, although it should be possible to install scales or infrared beams that could signal to the BTU (and trigger an email alert) whenever the juice falls below a certain amount.

Many laboratory-based tasks use precise eyetracking equipment to detect eye movements as behavioral responses, yet this feature is not currently available in the source code of the Mymou system. Since the system already constantly acquires video feed of the NHPs for the facial recognition module, it might be feasible to gain some online eye position information. However, the low sampling rate of the onboard camera (30 fps) is likely insufficient for precise real-time eyetracking.

If a tablet device fails, there is no guarantee that the same tablet will still be available for purchase. This might require the holder to be adjusted to fit the new tablet dimensions, and might also require changes to the Android operating system. In the former case, thanks to the modular design of the tablet holder, installing a tablet with different dimensions only involves replacing one removable piece of plastic, costing approximately £50. The latter case might require some time in order to adapt the system’s code, although this might also be unnecessary, as we and other user groups continue to develop the Mymou system (for use with other tablets) and update the scripts on our Github page accordingly.

### Further applications

Because the Mymou system dispenses fluids, it can also be used to accurately monitor daily fluid intake. In standard NHP housing environments, daily water is dispensed via water spouts connected to either mains water or large bottles. This design has several disadvantages. First, water bottles can be dislodged from their holder, and/or the spouts may leak water, meaning that NHPs might not have access to their daily requirements. Second, unless NHPs are separated when fluid is available, it becomes nearly impossible to quantify how much fluid each animal consumes. For animals on fluid control protocols, accurate recording of fluid intake is an important welfare consideration, to ensure that NHPs receive their daily fluid requirements and that NHPs are growing within established growth curves (Prescott et al., 2010). The Mymou system addresses these issues by also being a water dispenser. By training NHPs to simply press and hold the tablet for water, the system (via real-time facial recognition) can track exactly how much water has been delivered to each NHP, and therefore can eliminate the need to separate NHPs for fluid administration. This also alleviates the need for staff to separate NHPs and administer water, which can be problematic on weekends or holiday periods. If power were lost to the system, there is a risk that water might not be accessible to the NHPs. As such, fluid scheduling via the Mymou system still needs human monitoring; the system can be set up to send out an email following successful water delivery, and the absence of this email update would signal the need for human attention.

The Mymou system allows for training prior to migrating to classical laboratory-based settings. However, using the Mymou system in conjunction with wireless recording technologies could allow full experiments to be performed outside the conventional laboratory setting. A number of wireless systems are being developed for freely moving NHPs (Capogrosso et al., 2016; Fernandez-Leon et al., 2015; Schwarz et al., 2014; Yin et al., 2014) that could receive signals from the Mymou system, which would allow for synchronization of behavioral events with neuronal activity during more naturalistic and freely moving conditions. For example, the Arduino board could produce a regular timing signal that is detected by both the tablet and the wireless recording system in order to synchronize the two devices, although the exact timing accuracy of this would need to be carefully examined. This is a focus of our current research, and we welcome any collaborations on such a project.

In summary, the Mymou system is the first low-cost, wireless, open-source, fully automated in-cage training system that provides accurate data logging without either the implantation of microchips or labor-intensive post-hoc analyses. It allows NHPs to learn complex behavioral tasks across the entire day and on weekends, all without a need to separate NHPs from their peer group or for experimenter intervention. The system provides major refinements over laboratory and other home cage-based systems and provides a platform for wireless electrophysiological investigations in more naturalistic, freely moving environments.

#### Author note

We are grateful to Sean Cavanagh, Lianne McCombe, Shirley Mark, and Tim Behrens for their assistance with the project. This work was supported by a Wellcome Trust New Investigator Award to S.W.K. (096689/Z/11/Z). The authors declare no competing financial interests.
